# Influence of Li concentration on structural, morphological and electrochemical properties of anatase-TiO_2_ nanoparticles

**DOI:** 10.1038/s41598-024-61985-5

**Published:** 2024-05-16

**Authors:** Thanin Putjuso, Sasitorn Putjuso, Attaphol Karaphun, Ekaphan Swatsitang

**Affiliations:** 1 Department of General Education (Physics and Mathematics), Faculty of Liberal Arts, Rajamangala University of Technology Rattanakosin, Wang Klai Kangwon Campus, Hua Hin , Prachuap Khiri Khan 77110 Thailand; 2 Department of Physics, Faculty of Science, Khon Kaen University, Khon Kaen, 40002 Thailand; 3 Institute of Nanomaterials Research and Innovation for Energy (IN-RIE), Khon Kaen University, Khon Kaen, 40002 Thailand

**Keywords:** Lithium-doped anatase TiO_2_ nanoparticles, Sol–gel method, Electrochemical properties, Supercapacitor electrodes, Electronic devices, Electronic properties and materials, Electronic devices

## Abstract

Lithium-doped anatase-TiO_2_ nanoparticles (Li_x_Ti_1-x_O_2_ NPs, x = 0, 0.05, 0.10, 0.15 and 0.20) could be synthesized by a simple sol–gel process. X-ray diffraction (XRD) results displayed the tetragonal (space group: I41/amd) of polycrystalline TiO_2_ anatase phase. The spectroscopy results of Raman and FT-IR confirmed the anatase phase of TiO_2_ through the specific modes of metal oxides vibration in the crystalline TiO_2_. Surfaces micrographs by scanning electron microscope (SEM) of agglomerated Li_x_Ti_1-x_O_2_ NPs showed a spongy like morphology. Transmission electron microscope (TEM) illustrated a cuboidal shape of dispersed NPs with particle size distributed in a narrow range 5–10 nm. Bruanauer Emmett-Teller (BET) results showed the increased surface area of Li_x_Ti_1−x_O_2_ NPs with increasing Li content. Li_x_Ti_1-x_O_2_ NPs (x = 0.05–0.20) working electrodes illustrated a pseudocapacitive behavior with excellent electrochemical properties through the whole cycles of GCD test. Interestingly, Li_0.1_Ti_0.9_O_2_ NPs electrode illustrated a high performance in terms of maximum specific capacitance 822 F g^−1^ at 1.5 A g^−1^ in 0.5 M Li_2_SO_4_ electrolyte, with excellent capacitive retention 92.6% after 5000 cycles GCD test.

## Introduction

Supercapacitor (SC) has been demonstrated to be a beneficial gadget for advance energy storage device for present and forthcoming technology, because it can work superior than conventional capacitors in criteria of stability, capacity, and energy density. Indeed, the performance of SC device is significantly governed by the electrochemical properties of electrodes, which depend strongly on the electrode materials. In general, as suggested and reported by numerous research articles, the efficient electrode should have a huge surface area, high porosity with appropriate pore size distribution and coated on a high conducting substrate^1–4^. In some kind of SC, the chemical surface of electrode, directly correlate to the oxidation state of the materials, is considered to be an important factor that influence the electrochemical properties, as well. In addition, distinct electrodes morphology obtained by different synthesis conditions and processes can also affect the electrochemical properties of SC. However, when benchmark to battery, SC has some disadvantages of low energy density and output voltage instability. Therefore, much efforts have been driven to overcome these problems to push SC to be properly applied as a novel energy storage device in a wide scale^1–4^.

Currently, TiO_2_ nanoparticles (NPs) was deliberated as an excellently potential metal oxide to be applied for SCs electrode owing to its proper pseudocapacitive behavior, easy to synthesis, nontoxic, low cost and ecological friendliness^4–7^. However, TiO_2_ NPs generally show low electrical conductivity, which can block their performance and needed to be improved. So far, applications of TiO_2_ NPs doped with metal ions (Cu, Co, Ni, Li, Ag) have been widely investigated^4,8^, because lithium (Li) is considered to be one of the most promising elements for use as a dopant. It has been applied in a number of disciplines, including passivation layers in perovskite solar cells, lithium-ion batteries, and nanosensors^9^. Li is also in charge of transporting to TiO_2_ NPs and ejecting electrons. Furthermore, lithium ions are very mobile, and can be doped with TiO_2_ to improve the conductivity and to improve electron transmission when oxygen vacancies are passivated^8–10^. For example, Teimouri et al.^10^ synthesized Li-doped TiO_2_ films that could show significantly improved conductivity with faster charge transfer in planar perovskite solar cells. Golvari et al.^11^ prepared dye-sensitized solar cells on the mesoporous beads of Li-doped TiO_2_ with 7.48% improvement of the device performance. The work of Lakra et al*.*^12^ confirmed a good capacitive behavior of synthesized TiO_2_ NPs and suggested the materials for SC electrodes application. Moreover, Wang et al*.*^13^ suggested that the faradaic storage behavior of nanocrystalline anatase TiO_2_ in aqueous electrolyte might be contributed to the conversion between Ti^4+^ and Ti^3+^ in the redox reaction. Meanwhile, the electrochemical properties of a symmetric hybrid SC with electrode of hydrothermally obtained SWCNTs/TiO_2_ had been studied by Lal et al*.*^14^, and the device delivered a high capacitance of 144 F g^−1^ with 20 Wh kg^−1^ of energy density and outstanding capacity retention of 95% after 50,000 cycles test. He et al*.*^15^ fabricated a current collector of SC based on TiO_2_ nanotube arrays (NTA) with a cathode of composite MnO_2_/TiO_2_ NTA, and attended a high capacitance of 1051 F cm^−2^. Moreover, a value of 608.2 F cm^−2^ was accomplished in case of using Fe_2_O_3_ modified TiO_2_ NTA as an anode, and the assembled asymmetric SC could retain about 91.7% capacitance after 5000 cycling tests. Another interesting material that had been considered as a promising applicant for efficient SCs electrode was heterostructure Co_3_O_4_/m-NTAs. In this study, Yu et al*.*^16^ reported a maximum value of 662.7 F g^−1^ for specific capacitance with retain 86.0% of the value after 4000 cycles test at 10 A g^−1^. Similarly, in the report of Li et al*.*^17^, an assembled symmetric solid-state SC based on TiO_2_-CNT electrodes could show a high value of 82.5 Wh kg^−1^ for energy density and 345.7 F g^−1^ at 1.0 A g^−1^ for specific capacitance. In addition, this TiO_2_-CNT SC could demonstrate a good cycling stability of 93.3% for 10,000 cycling test, which might be due to the fast ion diffusion on surface of the anatase structure. Furthermore, Kumar et al*.*^18^ found that a SC with carbon-supported TiO_2_ electrode could exhibit a specific capacitance 277.72 F g^−1^ at 25 mV s^−1^ in 1 M Na_2_SO_4_ aqueous electrolyte. In addition, Elshahawy et al*.*^19^ reported a value of 57.62 mF/cm for specific capacitance in 2 M KOH electrolyte of TiO_2_ nanorod arrays based SCs, and could retain a capacity of 91% at the 10000th cycle of test. Ojha et al*.*^20^ obtained the mesoporous Mn-doped TiO_2_ by a simple sol–gel and a solvothermal method. According to this work, they suggested that the enhanced electrochemical properties could be mainly ascribed by many factors such as a larger surface area, a mesoporous structure and an appropriate concentration of Mn doping that could lead to the improved conductivity of a wide band gap TiO_2_ NPs*.* Additionally, in the work of Hodaei et al*.*^21^, nitrogen-doped TiO_2_ NPs was obtained by a sol–gel process. The electrochemical study showed the enhanced capacitive performance, including a value of 311 F g^−1^ at 1 A g^−1^ for specific capacitance with remained 98.9% of the value after 4000 cycling tests. Regarding to the work of Hodaei et al*.*^21^, the authors reported that a sol–gel method was an efficient process for preparing pure and metal-doped TiO_2_ NPs to be applied for SCs electrode due to its unique chemical reaction that could yield high quality NPs. In addition, NPs of highly homogeneous size distribution with high surface area and very high purity (99.99%) could be obtained at a low temperature. Thus, a sol–gel synthesized metal-doped TiO_2_ NPs is expected to play an important role for increasing electrical conductivity that can further improve the whole capacitive performance of supercapacitor^22,23^.

Therefore, in this work, we focus on the electrochemical performance investigation of the as synthesized Li-doped anatase TiO_2_ NPs (Li_x_Ti_1-x_O_2_ NPs, x = 0, 0.05, 0.10, 0.15 and 0.20) obtained by a sol–gel process. Interestingly, all Li_x_Ti_1-x_O_2_ NPs (x = 0.05–0.20) electrodes could demonstrate a pseudocapacitive behavior with a high specific capacitance and good cycling stability compared to undoped sample. To the best of our knowledge, it is the first time to fabricate SC with electrodes based on Li-doped TiO_2_ NPs.

## Experimental

### Chemicals

Sigma Aldrich supplied titanium (IV) isopropoxide (C_12_H_28_O_4_Ti, 99.95%), lithium hydroxide (LiOH, 99.50%), polyethylene glycol, ammonium hydroxide (NH_4_OH, 99.50%), acetylene black (99.99%), polyvinylidene fluoride (PVDF) and ethanol (C_2_H_5_OH, 99%). Lithium sulfate (Li_2_SO_4_, 95%) is a product of Ajax Fine Chem Laboratory Chemicals. N-methyl-2-pyrrolidone (NMP, 99.5%) was obtained from RCI Labscan.

### Synthesis of pure and Li-doped anatase TiO_2_ NPs

In the process of synthesis pure anatase TiO_2_ NPs, 5 ml polyethylene glycol was added to a well stirred deionized (DI) water:ethanol solution with 4:1 ratio by volume (40:10 ml). Then 10 ml of C_12_H_28_O_4_Ti was gradually dropped to this solution, while vigorously stirred at room temperature on a magnetic stirrer hot plate for further 20 min. Then, 2.5 wt.% aqueous ammonia (NH_4_OH) was added dropwise to carefully controlled the pH at 7, and further stirring for 30 min. After that, increased the temperature of a solution to 60 °C and kept on stirring until a wet gel was formed, and allowed to dry at 75 °C. The final product was achieved by crushing the dried gel, ground to fine powder and pyrolized at 500 °C in a furnace for 2 h, using a heating rate of 2 °C/min. Li_x_Ti_1-x_O_2_ NPs (x = 0.05, 0.10, 0.15 and 0.20) was synthesized by a similar way, only that lithium hydroxide (LiOH) of 0.05, 0.10, 0.15 and 0.20 by wt % was added in the mixture solution before adding NH_4_OH.

### Electrodes fabrication for electrochemical properties study

The electrode slurries of Li_x_Ti_1-x_O_2_ NPs (x = 0, 0.05, 0.10, 0.15 and 0.20) were prepared by ball milling each product with PVDF and acetylene black at 80: 10: 10 wt% ratio in 500 µL NMP solvent at RT for 24 h. Each electrode was fabricated by dripping an active mass slurry of approximately 200 µL to coat on an area 1 cm^2^ at one end of ultrasonically cleaned nickel foam sheet of size 1 × 2 cm^2^, and dried for 2 h at 80 °C. After that, all electrodes were pressed at 1.5 tons for 1 min, and immersed in 0.5 M Li_2_SO_4_ aqueous electrolyte prior to electrochemical properties testing. The CV study was performed in an applied voltage of 0.0 to + 0.5 V at scan rate 10, 20, 30, 50, 100 and 200 mV s^−1^. The GCD study was performed at applied current density 1.5, 2, 4, 6, 8, 10 and 15 A g^−1^. The capacity retention was evaluated at the 2000th cycle of GCD test at 10 A g^−1^. The GCD results were used for the calculation of specific capacitance (C_s_) using Eq. (1)^24^,

1 $${C}_{s}=\frac{I\Delta t}{m\Delta V}$$

where *I*, *Δt,* *m,* and *ΔV* stand for the constant discharge current (A), discharge time (s), mass of active material in electrode (g) and potential window (V), respectively.

Additionally, the energy density (*E*_*sd*_) and power density (*P*_*sd*_) of electrodes were determined from the GCD results, using Eqs. (2) and (3)^24^, respectively.

2 $${E}_{sd}=\frac{{C}_{s}\times \Delta {V}^{2}}{7.2}$$

3 $${P}_{sd}=\frac{{E}_{sd}\times 3600}{\Delta t}$$

## Characterizations

X-ray source with CuKα (λ = 1.5406 Å) generated by X- ray diffractometer (Philips X’Pert) was used for crystal structure and phase identification of the products. Raman study using a laser of 532 nm excitation (DXR Smart, Thermo Scientific) was employed for TiO_2_ phase verification. Moreover, in order to confirm the existence of various modes of vibration between Ti and O bonding in the TiO_2_ crystalline structure, Fourier transform infrared spectroscopy (FTIR, Bruker, Senterra) was performed. The surface morphology inspection of products and particles size determination were accomplished by field emission scanning electron microscope (FE-SEM, FEI, Helios NanoLab G3 CX). In addition, the quantitative estimation for major elements in wt% of the products could be achieved using energy dispersive X-ray spectroscopy (EDS) with elemental mapping to display the distribution of elements. Furthermore, high magnified bright field images with selected area electron diffraction (SAED) patterns by transmission electron microscope (TEM, FEI, TECNAI G2 20) was performed for clearer observed products morphology and more accurate particles size determination, including phase and structure confirmation. An instrument of Autosorb1-Quantachrome was employed for the study of specific surface area and a type of pore distributed in samples through the Bruanauer Emmett-Teller (BET) and Barrett-Joyner-Halenda (BJH) techniques, respectively. Finally, an equipment of Wuhan Corrtest Instruments Corp Ltd. (Model CS350 Potentiostat/Galvanostat) was used for electrochemical properties studies of all Li_x_Ti_1-x_O_2_ NPs electrodes to obtain the CV, GCD and EIS results.

## Results and discussion

**Figure 1:**
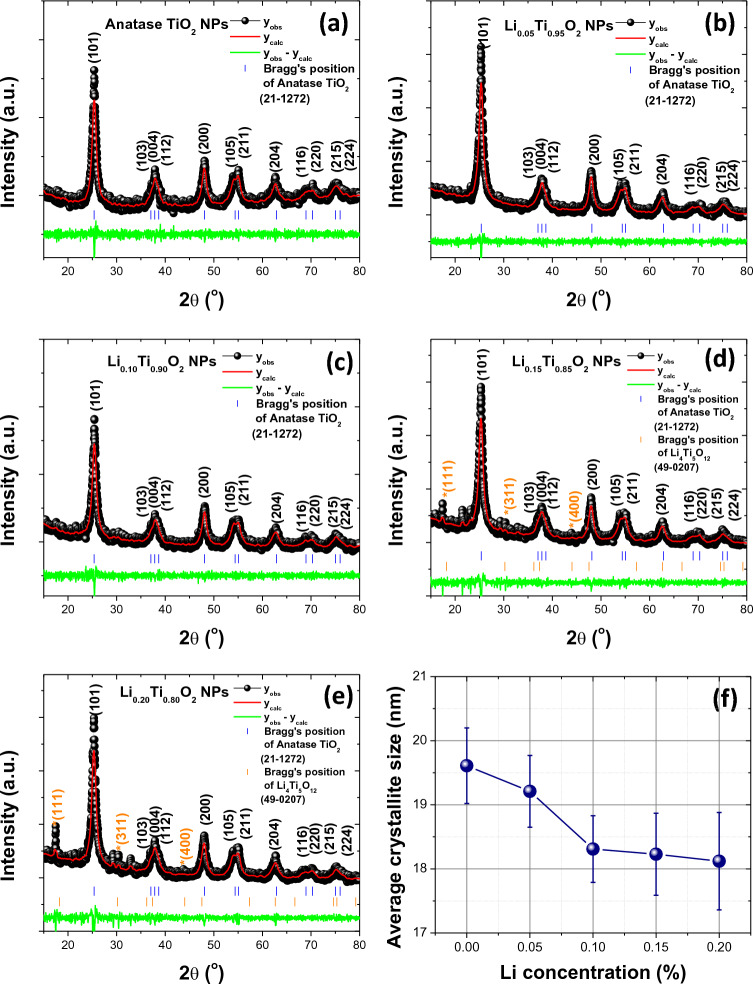
Rietveld refinement fitted XRD patterns of samples with different Li concentrations and plot of average crystallite size vs. Li concentration.

The XRD patterns with Rietveld refinement fitting of Li_x_Ti_1−x_O_2_ NPs are displayed in Fig. 1a–e. In Fig. 1a–e, the most dominant XRD peaks at 25.26°, 36.92°, 47.96°, 53.92°, 55.01°, 62.83°, 70.24° and 75.04° correspond to the crystalline diffraction plane (101), (004), (200), (105), (211), (204), (116), and (215), respectively. The XRD results matched with the standard data of JCPDS: 21–1272 for the tetragonal anatase TiO_2_ crystalline phase of space group: I41/amd^9,18,25^. However, in a sample of x = 0.15 and 0.20 (Fig. 1d, e), many peaks of monoclinic Li_4_Ti_5_O_12_ phase with space group: C2/c observed at 17.32°, 30.95° and 44.49° correspond with the diffraction plane (111), (311) and (400), respectively, and matching to the standard data of JCPDS: 49-0207. The formation of Li_4_Ti_5_O_12_ phase might be due to the direct interaction of excess Li with pure anatase crystalline TiO_2_ phase during the growth process. It was suggested that this phase could provide a nonsymmetric stretching vibration of O–Ti–O that could result in reduced conductivity of the samples. Moreover, the cell parameter (*a*, *b* and *c*) with cell volume and various parameters (R_wp_, R_p_, R_ex_ and GOF, definition for these parameters was given elsewhere) were evaluated by Rietveld refinement method using a standard data of JCPDS: 21-1272 (tetragonal phase with space group: I41/amd) and JCPDS: 49-0207 (monoclinic phase with space group: C2/c), as displayed in Fig. 1a–e, and summarize of the results was listed in Table 1. As seen in Table 1 and the excellent fitting of the XRD patterns in Fig. 1a–e, it can be concluded that Li loading significantly affect the cell parameters of anatase TiO_2_ phase. Obviously, the cell parameters and cell volume of samples decrease with increasing Li loading, leading to the deceased crystallite size of Li_x_Ti_1−x_O_2_ NPs. Generally, Ti^4+^ in a unit cell of TiO_2_ crystal system is bonded to six equivalent O^2−^ atoms, leading to the formation of mixture distorted edge and corner-sharing TiO_6_ octahedra. Furthermore, in a unit cell of Li_4_Ti_5_O_12_ phase, a complicated structure is formed owing to the formation of LiO_4_ tetrahedra by the bonding of Li^1+^ with four O^2−^ atoms at the cell corners that could be shared with others two equivalent LiO_6_ octahedra and ten TiO_6_ octahedra. Moreover, the percentage of anatase TiO_2_ phase and Li_4_Ti_5_O_12_ phase were determined in samples of x = 0.15 and 0.20, and found to be (95.12 and 4.88%) and (92.23 and 7.77%), respectively. Additionally, the X-ray line of the diffraction planes (101), (004), (200), (105), (211), (204), (116), and (215) were used for the evaluation of average crystallite sizes (*D*_*Sh*_) of all samples, using the Scherrer’s equation (4).

**Table 1:** Cell parameters and phase analysis investigated by XRD and Rietveld refinement fitting with the results of surface and pore analysis of Li_x_Ti_1−x_O_2_ NPs.

Parameter		Anatase TiO_2_ NPs	Li_x_Ti_1-x_O_2_ NPs					
			x = 0.05,	x = 0.10,	x = 0.15		x = 0.20	
Space group		I41/amd	I41/amd	I41/amd	I41/amd	C2/c	I41/amd	C2/c
Crystal structure		Tetragonal	Tetragonal	Tetragonal	Tetragonal	Monoclinic	Tetragonal	Monoclinic
Crystalline parameter (Å)	*a*	3.78(3)	3.78 (3)	3.78(4)	3.77(4)	8.33(2)	3.77(2)	8.34(4)
	*b*	3.78 (2)	3.78 (2)	3.78 (4)	3.77(3)	8.33(2)	3.77(2)	8.34(4)
	*c*	9.51(3)	9.51(2)	9.50(4)	9.50(3)	13.14(4)	9.50(2)	13.17(1)
α = γ = β (°)		90	90	90	90	α = γ (90) β(108.1)	90	α = γ (90) β(107.9)
Cell volume (10^6^ pm^3^)		135.88	135.42	135.34	134.77	872.13	134.10	872.66
R_ex_ (%)		4.7331	5.3895	4.9934	5.2199		5.2424	
R_p_ (%)		4.5838	3.9797	4.0386	4.2830		4.6028	
R_wp_ (%)		6.0787	5.1291	5.2259	5.5440		6.0595	
GOF		6.0973	4.1996	3.1963	4.6255		4.1498	
*D*_*Sh*_ (nm)		19.61 ± 0.59	19.21 ± 0.56	18.31 ± 0.52	18.23 ± 0.64		18.12 ± 0.76	
% phase of TiO_2_ and Li_4_Ti_5_O_12_		100	100	100	95.12 4.88		92.23 7.77	
*D*ps (nm)		30.04 ± 4.92	27.97 ± 6.56	25.12 ± 2.64	25.03 ± 5.53		24.66 ± 6.13	
Specific surface area (m^2^ g^−1^)		163.01	180.23	246.94	239.92		241.93	
Average pore-size (nm)		6.54 ± 0.93	6.31 ± 0.76	5.91 ± 0.66	5.71 ± 0.58		5.80 ± 0.61	
Pore volume (cm^3^ g^−1^)		0.30	0.31	0.35	0.37		0.38	

4 $$DSh =k\lambda/\beta \cos\theta ,$$

In Eq. (4), the parameters *θ*, *λ* and *β* are defined for Bragg angle, wavelength of X-ray and full width at half maximum, respectively. *k* is the constant and was taken as 0.9. The evaluated *D*_*Sh*_ values are 19.61 ± 0.59, 19.21 ± 0.56, 18.31 ± 0.52, 18.23 ± 0.64 and 18.12 ± 0.76 nm for Li_x_Ti_1-x_O_2_ NPs of x = 0, 0.05 0.10, 0.15 and 0.20, respectively. All the *D*_*Sh*_ values were summarized in Table 1, and the plot of *D*_*Sh*_ versus Li concentration is illustrated in Fig. 1f. Obviously seen in Fig. 1f, *D*_*Sh*_ decreases with increasing Li concentration. The decreased *D*_*Sh*_ was suggested to originate from the replacement of a large ionic radius of Ti^4+^ (0.745 Å) and Ti^3+^ (0.670 Å) by a small ionic radius of Li^+^ (0.60 Å) in the anatase TiO_2_ crystal structure. By comparing the ionic radius of Li^+^ (0.60 Å) and Ti^4+^ (0.745 Å), it is clear that a possible substitution of a small amount of Ti^4+^ by Li^+^ would be accompanied by a weak lattice expansion, due to the relatively small difference between their respective ionic radius, so that the Li^+^ ions can be dissolve into anatase TiO_2_ phase and Li_4_Ti_5_O_12_ phase^26^. However, the substitution might induce lattice expansion, resulting in a shift of the anatase peak to the lower angles. Although for the replacement of Ti^4+^ by L^i+^ ions, some Ti–O bonds are broken, which leads to the formation of oxygen vacancies, the contraction of lattice caused by oxygen deficiency is eliminated through lattice expansion induced by the presence of the slightly smaller lithium ions^11^. As a result, Li^+^ ions appear to be an appropriate option for modifying the local crystal structure at Ti^4+^ sites in TiO_2_, because they could operate as charge compensators and could additionally enhance capacitive properties due to the availability of more active sites^27^.

**Figure 2:**
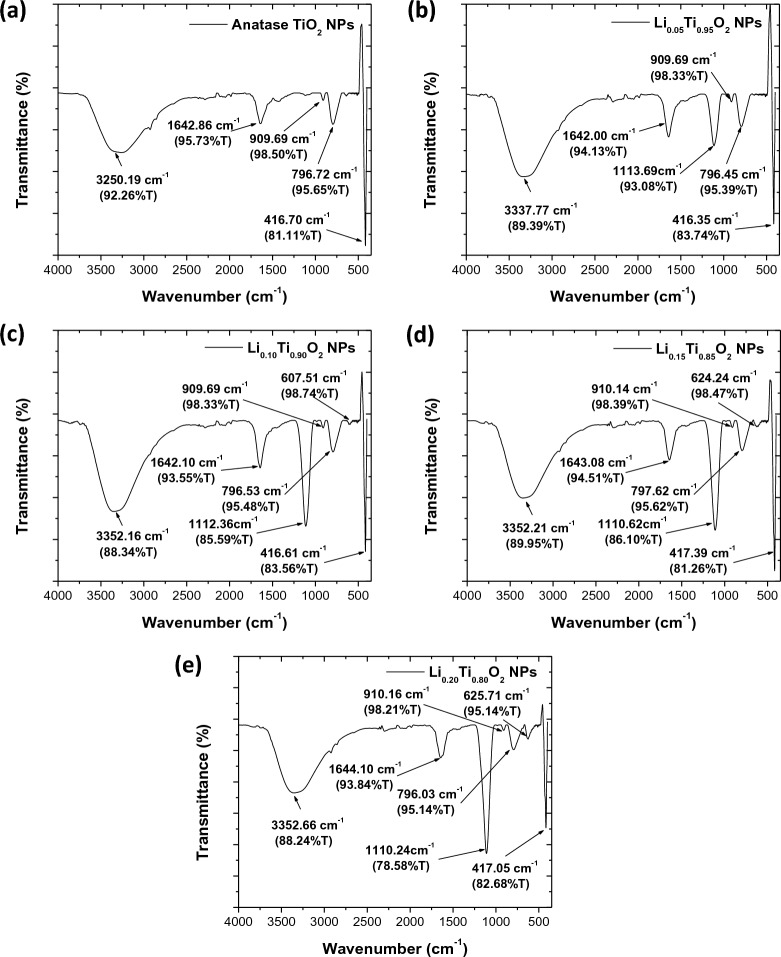
FTIR results of Li_x_Ti_1−x_O_2_ NPs with different Li concentrations.

FTIR spectra of samples are displayed in Fig. 2a–e. The broaden vibration peaks around 3250–3350 cm^−1^ are assigned for O–H stretching modes, relating to the stretching vibration of the hydroxyl (O–H) group due to the formation of H_2_O molecules on surface^11^. Moreover, the appeared vibration peaks around 1640–1644 cm^−1^ are designated to the symmetric stretching of Ti–OH on surface^18^. Additionally, the observed peaks around 1110–1113 cm^−1^ and 600–625 cm^−1^ indicated the bonding of Ti–O in an anatase TiO_2_ structure^11^. In the samples with x = 0.10, 0.15 and 0.20, the observed peaks in a range 790–900 cm^−1^ are attributed to the symmetric C–H and asymmetric CH_2_ vibrations of an organic polyethylene glycol that could not be completely removed after calcination^11^. The strong vibration peaks in a range 410–625 cm^−1^ are associated with the vibration modes of O–Ti–O bonding in an anatase TiO_2_ structure.

**Figure 3:**
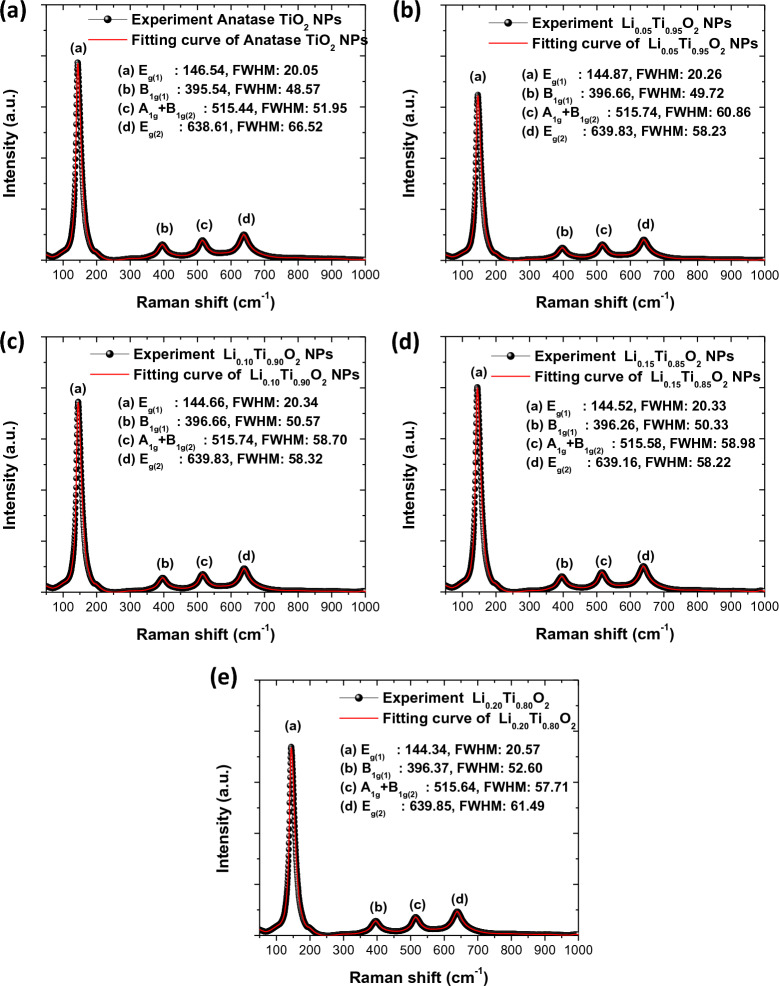
Raman results of Li_x_Ti_1−x_O_2_ NPs with different Li concentrations.

Further structural analysis of Li_x_Ti_1−x_O_2_ NPs (x = 0, 0.05, 0.10, 0.15 and 0.20) was performed by Raman technique, as shown in Fig. 3a–e. In these figures, the major sharp Raman shift observed at ~ 144 cm^−1^ corresponds to the E_g(1)_ mode of anatase TiO_2_^18,28^. The peak at 396 cm^−1^ corresponds to the B_1g(1)_ mode, while another at 639 cm^−1^ corresponds to the E_g(2)_ mode, arising from the symmetric stretching mode of O–Ti–O bonding in crystallite anatase TiO_2_^28^. The other one observed at 515 cm^−1^ was assigned for A_1g_ + B_1g(2)_ mode, corresponding to the antisymmetric bending vibration of O–Ti–O bonding in TiO_2_ structure^18,28^. Therefore, Raman results confirmed the anatase phase of all samples.

**Figure 4:**
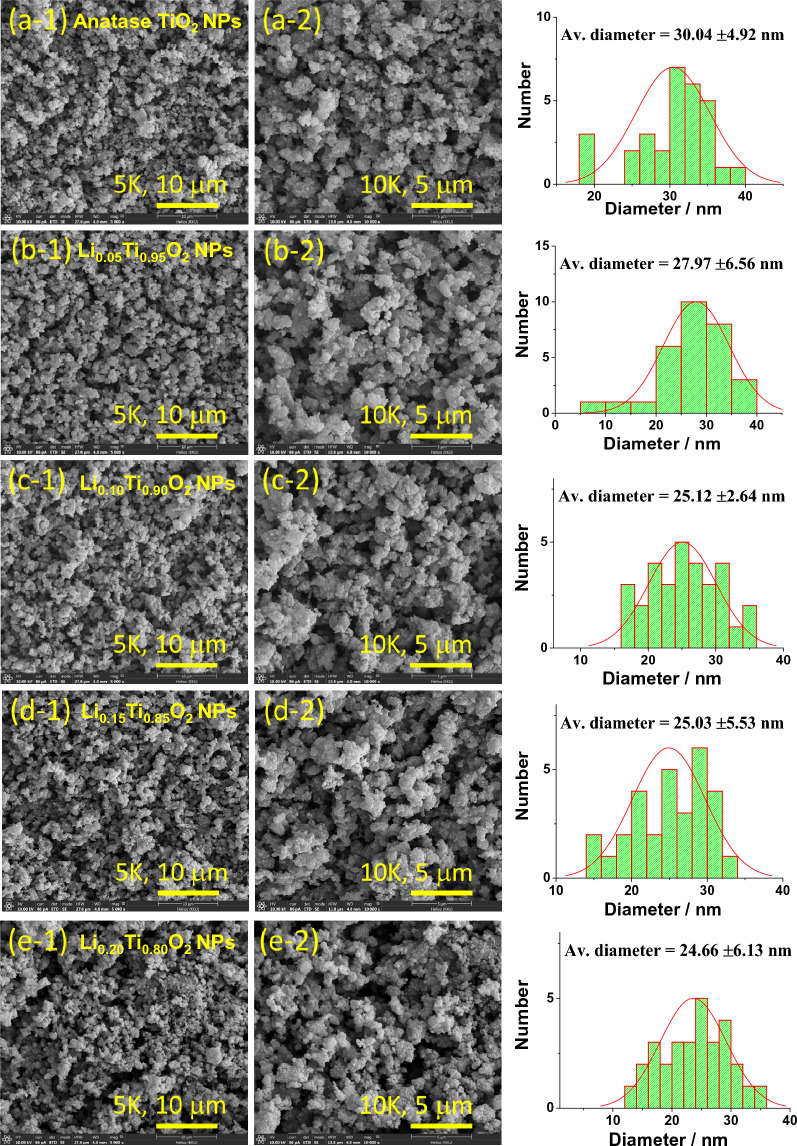
FE-SEM images (×5000  and ×10,000  magnification) and histograms for particles size distribution of Li_x_Ti_1−x_O_2_ NPs.

**Figure 5:**
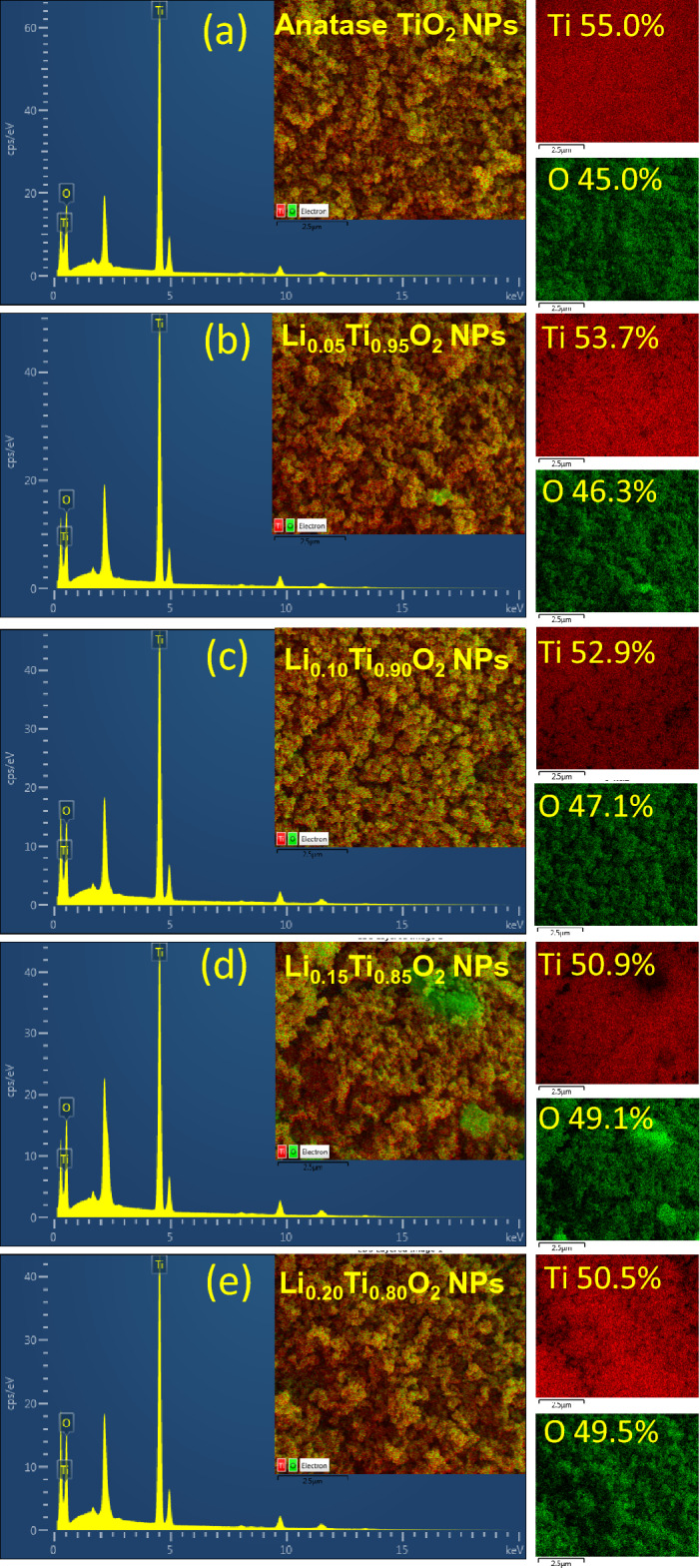
EDS results and mapping images of Li_x_Ti_1−x_O_2_ NPs.

Morphology and average particles size (*D*ps) of Li_x_Ti_1−x_O_2_ NPs are demonstrated by FE-SEM images with corresponding histograms in Fig. 4a–e. All images show the homogeneous distribution of agglomerated NPs with intercalated space between them, illustrating the micrographs of porous surface similar to spongy materials. However, a secondary monoclinic phase of Li_4_Ti_5_O_12_ NPs that existed in the samples with x = 0.15 and 0.20 could not be observed or identified by SEM micrographs in Fig. d-1, d-2, e-1, e-2 was suggested to be owing to the small amount of them compared to a major TiO_2_ phase, as estimated and listed in Table 1. The *D*ps values are 30.04 ± 4.92, 27.97 ± 6.56, 25.12 ± 2.64, 25.03 ± 5.53 and 24.66 ± 6.13 nm for samples of x = 0, 0.05 0.10, 0.15 and 0.20, respectively. The *D*ps values were listed in Table 1. As seen in Table 1, *D*ps decreases with increased Li loading. The uniform dispersion of agglomerated TiO_2_ NPs in electrodes could lead to enhance the porosity and form the conducting networks for charge transfer, as suggested by Prashad et al*.*^20^. Moreover, the observed porous structure of Li-doped anatase TiO_2_ NPs could increase the surface area of the electrode materials for the adsorption of electrolyte ions, resulting in more charges collection and could finally enhance the specific capacitance. Figure 5a–e display the EDS results and mapping of elements for Li_x_Ti_1−x_O_2_ NPs. The EDS results clearly show the uniform distribution of major elements Ti and O in the samples. However, Li element could not be detected due to its light-weight and a limitation of the instrument. The atomic percentages for Ti element were estimated to be about 55.0%, 53.7%, 52.9%, 50.9% and 50.5% for Li_x_Ti_1−x_O_2_ NPs with x = 0, 0.05 0.10, 0.15 and 0.20, respectively. The decreased amount of Ti was due to the Li replacement in anatase crystal structure of TiO_2_.

**Figure 6:**
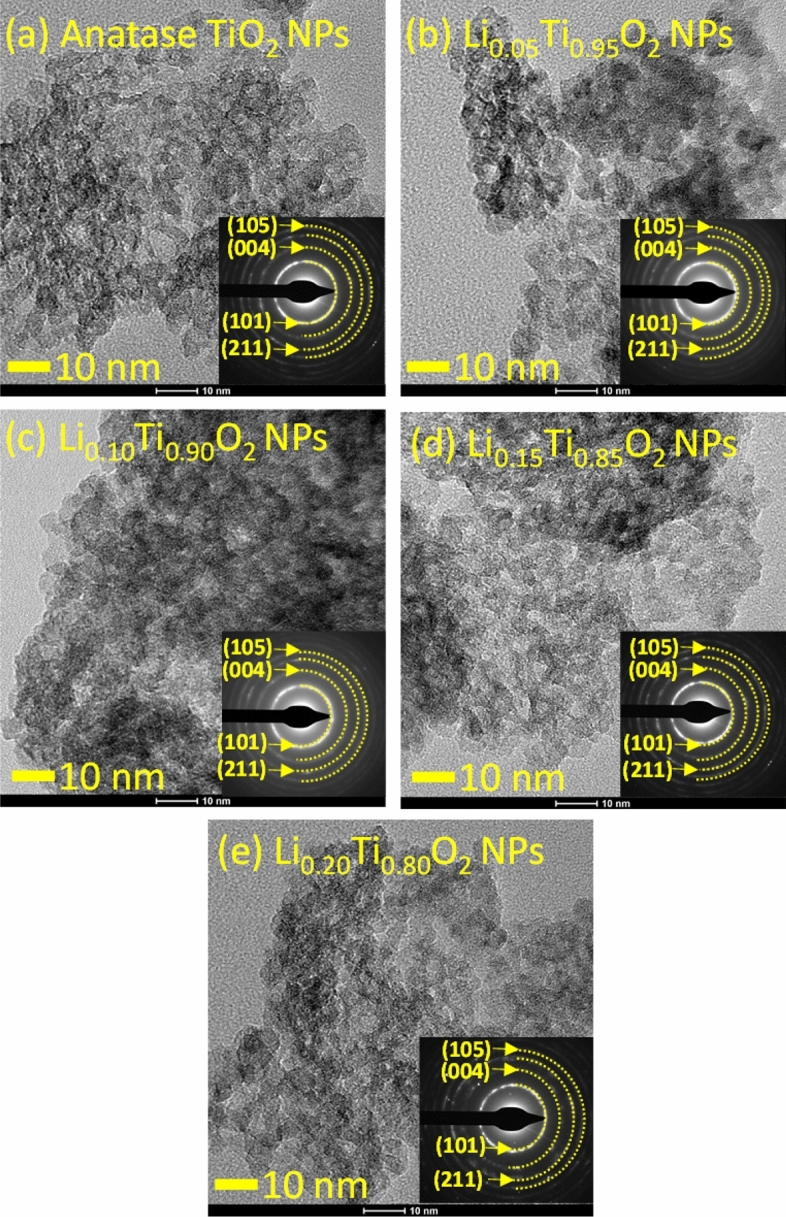
TEM bright field images with insets showing indexed SAED patterns of Li_x_Ti_1−x_O_2_ NPs.

In fact, more accurate particles size of Li_x_Ti_1-x_O_2_ NPs can be evaluated from TEM bright field images, including a better clear morphology of particles, as illustrated in Fig. 6a–e. As obviously seen, all images display NPs of very fine cuboidal shape with irregular size and agglomerated to form a spongy like-structure with roughly estimated individual particle size in an interval of 5–10 nm. Notably, the particle sizes estimated by TEM are smaller than those evaluated by SEM, which might be due to the dispersion of NPs during the sonication process of samples preparation prior to TEM performance. Moreover, the median size of NPs could be slightly reduced with the inclusion of Li^+^ ions on the Ti^4+^ and Ti^3+^ sites in the anatase TiO_2_ crystalline structure^18,20^. Furthermore, the unique morphology and homogenous size distribution in a narrow range of TiO_2_ NPs was suggested to result in the increase of materials porosity and surface area. Additionally, the halo ring shape with arranged spots on the circumferences of SAED patterns in all samples indicate a polycrystalline nature of the materials^18^. Moreover, all SAED patterns had been indexed to be composed of different crystalline planes that correspond to those of anatase phase TiO_2_, agreeing well with the XRD results shown in Fig. 1.

**Figure 7:**
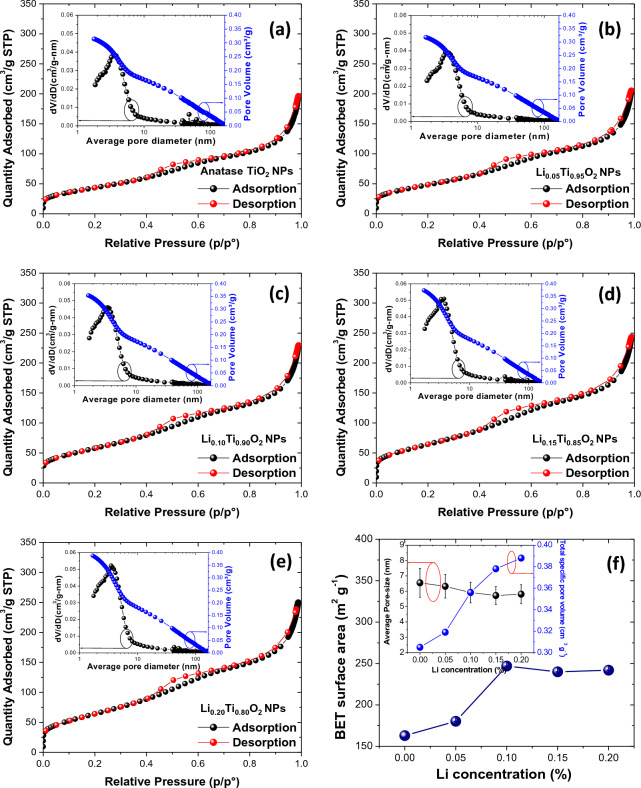
(**a–e**) Nitrogen sorption isotherms results with inset showing average pore diameter of Li_x_Ti_1−x_O_2_ NPs. (**f**) Plots of BET surface area, average pore size and total specific pore volume (inset) as a function of Li concentration.

Figure 7a–e display the N_2_ adsorption/desorption isotherms results of Li_x_Ti_1-x_O_2_ NPs. Regarding to these results, the observed hysteresis loops of all samples exhibit the BET curve of type IV, corresponding to that of mesoporous materials^11,20^. The evaluated specific surface area of anatase TiO_2_ NPs (x = 0) was 163.01 m^2^ g^−1^, whereas those of Li_x_Ti_1-x_O_2_ NPs (x = 0.05, 0.10, 0.15 and 0.20) displayed the larger values of 180.23, 246.94, 239.92 and 241.93 m^2^ g^−1^, respectively. Moreover, the average pore size and total specific pore volume of Li_x_Ti_1−x_O_2_ NPs (x = 0, 0.05, 0.10, 0.15 and 0.20) by the BJH technique were found to be 6.54 ± 0.93, 6.31 ± 0.76, 5.91 ± 0.66, 5.71 ± 0.58 and 5.80 ± 0.61 nm, and 0.30, 0.31, 0.35, 0.37 and 0.38 cm^3^ g^−1^, respectively. All of these values were listed in Table 1, and their plots as a function of Li concentration are illustrated in Fig. 7f. It was suggested that the great increase of specific surface area with small pore size of Li-doped anatase TiO_2_ NPs could create the appropriate pathways for ions to diffuse into the surface of electrodes, resulting in the increased charge collection and improvement of the capacitive performance^11,20^. The morphology and pore size of the produced compounds indicated nanoscale particles that could provide high electrolyte–electrode interfacial surface area, resulting in the comprehensive permeation of electrolyte and minimizing the path of transport to accelerate the fast transfer of Li^+^ and e^−^ in Li-doped anatase TiO_2_ NPs cathode^27^. Furthermore, the mesoporous nature of electrode could improve the access of electrolyte into the bulk of the materials, while also providing high power tapping densities and robust structural and electrical interconnectivity across the electrode^29^.

**Figure 8:**
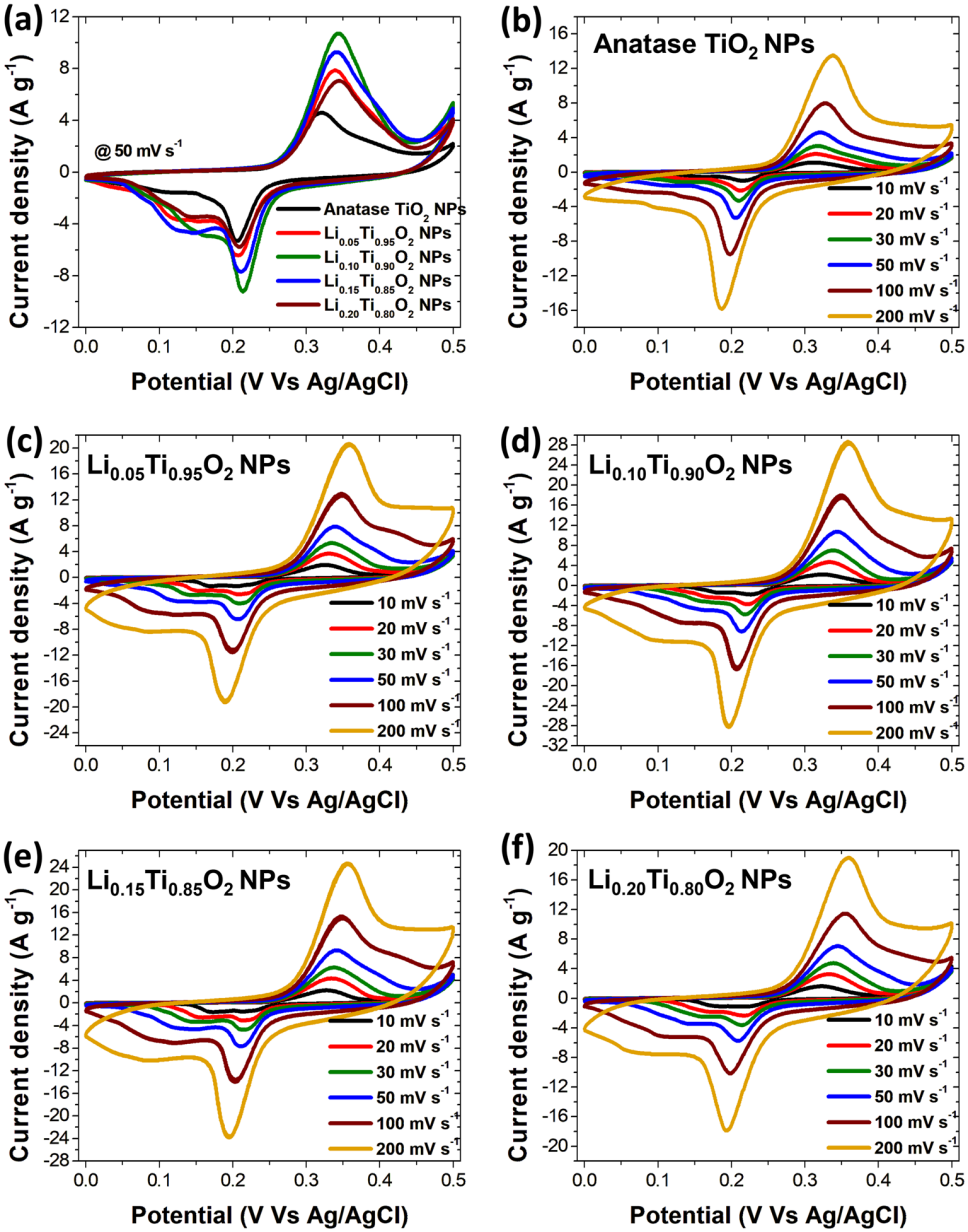
CV curves of Li_x_Ti_1−x_O_2_ NPs electrodes.

Cyclic voltammetry (CV) measurements of Li_x_Ti_1−x_O_2_ NPs (x = 0, 0.05, 0.10, 0.15 and 0.20) electrodes were performed at a scan rate 50 mV s^−1^ in 0.5 M Li_2_SO_4_ electrolyte within a potential window 0.0–0.5 V. The capacitive performance of each electrode is shown in Fig. 8a. The electrochemical performance of all electrodes performed at different scan rates are displayed in Fig. 8b–f. As seen in Fig. 8a, the CV curve of Li_0.10_Ti_0.90_O_2_ NPs electrode reveals the largest size, indicating a superior electrochemical performance compared to other electrodes. According to Fig. 8a–f, the distorted rectangular shape with apparent redox peaks of CV curves are observed, suggesting a typical pseudocapacitive behavior of Li_x_Ti_1−x_O_2_ NPs electrodes^4,16,20^. Moreover, all curves exhibit the stability with increasing scan rate through the whole applied voltage range^4,18,20^. Additionally, it is obviously seen that with enhanced potential sweep rate from 10 to 200 mV s^−1^, the anodic and cathodic peaks are shifted to the negative and positive values, respectively. In addition, the appeared anodic and cathodic peaks in a voltage range 0.18–0.35 V were due to the faradaic redox reaction of TiO_2_ NPs. Generally, the appearance of redox peaks is correlated to the cation interaction on the TiO_2_ surface, which can be expressed as^4,30^:

5 $$\left({\text{TiO}}_{2}\right)surface +\mathrm{ Li}+ +\mathrm{ e}- \leftrightarrow ({\text{TiO}}_{2}-\mathrm{ Li}+) surface$$

where Li^+^ could be the Li_2_SO_4_ electrolyte. For CV curves of Li_x_Ti_1−x_O_2_ NPs electrodes displayed associated with Li^+^ intercalation and de-intercalation into Li_x_Ti_1−x_O_2_ NPs electrodes. The overall cell reaction for the Li^+^ insertion/extraction into Li_x_Ti_1−x_O_2_ NPs electrodes can be written as^4,30^:

6 $$({\text{LixTi}}1-{\text{xO}}_{2}) surface +\mathrm{ Li}+ +\mathrm{ e}- \leftrightarrow ({\text{Lix}}({\text{Ti}}4+/{\text{Ti}}3+)1-{\text{xO}}_{2} -\mathrm{ Li}+) surface$$

Owing to the excellent ability of alteration between different oxidation states of Ti ions during the redox reaction, TiO_2_ was suggested to be a potential material for SCs electrode. Regarding to the redox reaction, Ti^4+^ was transferred to Ti^3+^ while charging, and converted to the initial state during discharging^18,19^, showing a pseudocapacitive behavior of n-type semiconductor for all Li_x_Ti_1-x_O_2_ NPs electrodes. In addition, it was reported that during discharge, a number of Li^+^ ions was inserted into the interstitial sites of the Li-doped TiO_2_ NPs framework, which implied a partial reduction of Ti^4+^ to Ti^3+^ state^29,31–34^. Moreover, it was also suggested that the redox reaction could be possibly affected by the thickness variation of diffusion layers in electrodes due to using different scan rates in the measurements. The obviously enhanced current with scan rates indicates a good rate capability of Li_0.10_Ti_0.90_O_2_ NPs electrode. While the conductivity of electrodes with higher Li concentration (x = 0.15 and 0.20) was observed to decrease, suggesting to be affected by the defect sites generated by the presence of Li_4_Ti_5_O_12_ phase in the materials.

**Figure 9:**
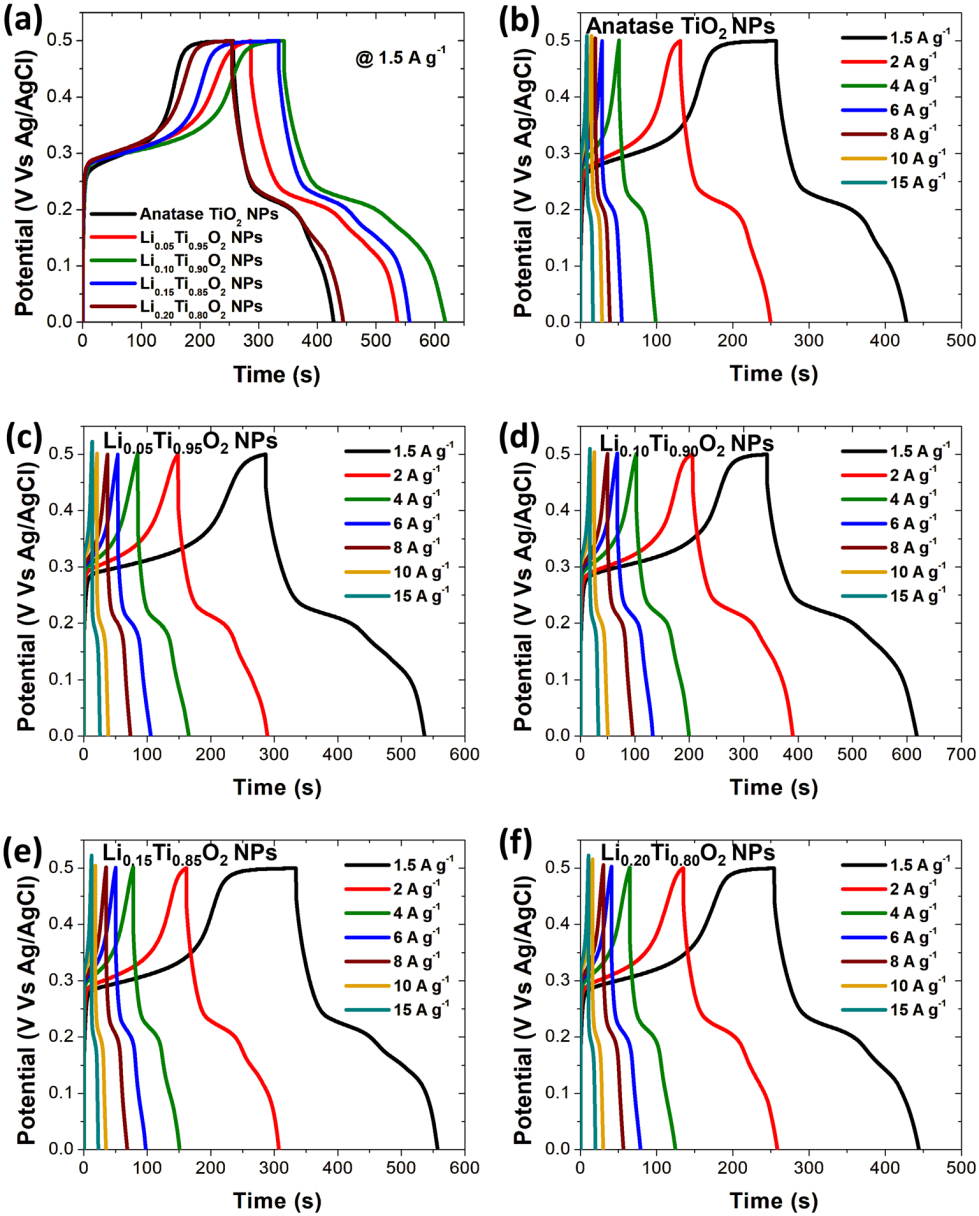
(**a**) GCD results of all Li_x_Ti_1−x_O_2_ NPs electrodes at 1.5 A g^−1^. (**b–f**) GCD results of all Li_x_Ti_1−x_O_2_ NPs (x = 0.0–0.20) electrodes performed at different current densities.

**Figure 10:**
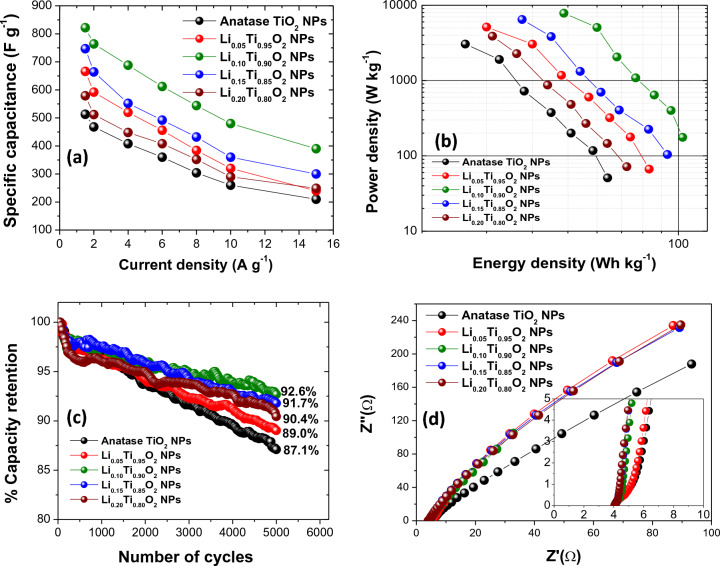
Electrochemical performance of all electrodes, (**a**) specific capacitance vs. current density, (**b**) Ragone plots, (**c**) cycling stability at 10 A g^−1^ and (**d**) Nyquist plots of all electrodes in aqueous electrolyte of 0.5 M Li_2_SO_4_.

For further investigation of electrochemical properties of Li_x_Ti_1−x_O_2_ NPs electrodes, the galvanostatic charge discharge (GCD) measurements were performed at 1.5 A g^−1^, and the results are displayed in Fig. 9a. In addition, more GCD results performed with variation of current density from 1.5 to 15 A g^−1^ for each electrode are displayed in Fig. 9b–f. In these figures, GCD curves of all electrodes illustrate a non-symmetrical voltage–time profile at each constant current density, indicating a pseudocapacitive behavior with superior electrochemical reversibility through the whole charge/discharge process^4,18,20^. The maximum specific capacitance (C_s_) at 1.5 A g^−1^ of 822 F g^−1^ was achieved in Li_x_Ti_1−x_O_2_ NPs electrode with x = 0.10, while the C_s_ values of others electrode with x = 0, 0.05, 0.15 and 0.20 were evaluated to be 513, 666, 747 and 579 F g^−1^, respectively, as displayed in Fig. 10a, and summarized in Table 2. The longer discharge time than other electrodes at 1.5 A g^−1^ (see Fig. 9b–f) suggests a good electrochemical performance of Li-doped TiO_2_ Nps electrode with 0.10% Li loading. The GCD profile of Li_0.10_Ti_0.90_O_2_ NPs electrode, displaying a good performance with the highest C_s_ value, is in good agreement with its CV results shown in Fig. 8a, d. Generally, *P*_*sd*_ and *E*_*sd*_ obtained by the Ragone plot are two other important parameters used for evaluating the supercapacitor performance. Accordingly, Fig. 10b displays the Ragone plots of all Li_x_Ti_1-x_O_2_ NPs electrodes, illustrating the decreased *P*_*sd*_ and *E*_*sd*_ values with increasing current density, which might be correlated to the limit of ions diffusion in electrodes and electrolyte^21^. The energy density (*E*_*sd*_) and power density (*P*_*sd*_) at 1.5 A g^−1^ of Li_x_Ti_1−x_O_2_ NPs electrodes (x = 0, 0.05, 0.10, 0.15 and 0.20) are (64.12, 83.25, 102.75, 93.38 and 72.37 W h kg^−1^), and (51.04, 66.67, 176.04, 104.16 and 51.04 W kg^−1^), respectively. As seen, all Li-doped anatase TiO_2_ NPs electrodes revealed the improved *P*_*sd*_ and *E*_*sd*_ as compared to that of undoped anatase TiO_2_ NPs electrode. The increased electrical conductivity and surface area of Li-doped anatase TiO_2_ NPs with increasing Li content were suggested for the improved performance. Furthermore, the GCD results for electrochemical stability study at 10 A g^−1^ with 5000 cycles test are illustrated in Fig. 10c. According to these results, a high capacitance retention of 87.1%, 89.0%, 92.6%, 91.7% and 90.4% at the 5000th cycles were attained for Li_x_Ti_1−x_O_2_ NPs electrodes with x = 0, 0.05, 0.10, 0.15 and 0.20, respectively, and the values were listed in Table 2. Obviously, all electrodes of Li-doped anatase TiO_2_ NPs illustrated a superior capacitance retention as compared to undoped anatase TiO_2_ NPs^18^. Actually, it has been observed that good stability after testing for 5000th cycles of all Li_x_Ti_1−x_O_2_ NPs electrodes in aqueous electrolyte of 0.5 M Li_2_SO_4_ is the most interesting one, since three main effects are evident; (i) decrease in the aggregation and overlapping of Li_x_Ti_1−x_O_2_ NPs during a long time required by the charge–discharge process, (ii) the fast Li^+^ ions diffusion and increased electronic conductivity on surface of Li_x_Ti_1−x_O_2_ NPs, and (iii) a slight shift of the CV and GCD curves is a very promising strategy to produce an environment friendly supercapacitor, which is able to reach in the future for the targeted energy and power density of organic electrolyte-based systems with acceptable good electrochemical performance^35–38^. Additionally, the EIS results of all Li_x_Ti_1−x_O_2_ NPs electrodes obtained in a range 0.01–100 kHz frequency at 5 mV are shown in Fig. 10d, and all the obtained data were provided in Table 2. Next, the series resistance (R_s_) of Li_x_Ti_1−x_O_2_, NPs electrodes with x = 0, 0.05, 0.10, 0.15 and 0.20 were determined to be 4.05, 4.03, 4.11, 4.10 and 4.03 Ω, respectively. Moreover, Li-doped anatase TiO_2_ NPs electrodes showed the low Rct value evaluated from a semicircle part in a high frequency region near the origin of the plots, meanwhile the plots connected to a low frequency region show almost the incline straight lines parallel to Z'' (Ω)^4,18,20,21^. As results, this might cause the formation of more vacancy sites in TiO_2_ lattice as well as the faster charge transferability in the Li-doped anatase TiO_2_ NPs electrodes, ascribed primarily due to the change of the intrinsic conductive properties of TiO_2_ NPs due to Li doping^18,20^. For Warburg resistance (W_R_), which describes the ion diffusion process of redox materials, the W_R_ values for all electrodes in a low frequency range were generally evaluated from the slopes of Z'' plots that abruptly increase in a straight line next to a high frequency region. As seen in Table 2, the W_R_ value of Li_x_Ti_1−x_O_2_ NPs electrode with x = 0.10 is lower than those of other electrodes. The lower value of W_R_ implies the faster ion transferability from electrolyte to electrode, resulting in a greater C_s_ value^18,20^. The EIS results clearly indicate the decreased series resistance of Li-doped anatase TiO_2_ NPs electrodes as compared with the bare anatase TiO_2_ NPs electrode. Regarding to the ascribed results, it was suggested that Li_x_Ti_1−x_O_2_ NPs with x = 0.10 was an appropriate material to be applied for electrode of high-performance supercapacitors. To express the excellent and attractive capacitive value of Li_0.10_Ti_0.90_O_2_ NPs electrode, the comparison of its electrochemical performance with others TiO_2_-based electrode was illustrated in Table 3. Therefore, in this study, we thoroughly explored the influence of Li-doped anatase-TiO_2_ NPs on high-performance supercapacitors. Generally, pentavalent donor-type doping is required to increase the material's electrical conductivity, and Li^+^ ions can easily substitute Ti^4+^ and Ti^3+^ ions in the anatase-TiO_2_ lattice at a wide range of concentrations. According to the experimental results, adding a modest amount of Li to the material could potentially affect many factors such as crystal size, phase purity, morphology, surface area, and pore size distribution. Consequently, Li_x_Ti_1−x_O_2_ NPs electrode with x = 0.10 exhibited superior ion diffusivity, high conductivity, and small particle size compared to the other samples. As a result, Li_0.10_Ti_0.90_O_2_ NPs electrode exhibited a higher electrochemical activity compared to the others electrode. The increased lattice parameters could improve its overall performance by enhancing its metallic-like character, while having minor effects on its electrochemical properties. The CV and GCD tests showed that the Li_0.10_Ti_0.90_O_2_ NPs electrode exhibited a pseudocapacitive storing mechanism, which occurred on the electrode surfaces at Li-doped Ti sites. As a result, Li^+^ ions played an important role in the improvement of electrical conductivity, charge storage capacity and stability with capacitance retention reaching as high as 92.6% after 5,000 cycles GCD test.

**Table 2:** C_s_ values and capacity retention with energy density, power density and EIS analysis of all Li_x_Ti_1−x_O_2_ NPs (x = 0.0–0.20) electrodes.

Parameter		Anatase TiO_2_ NPs (x = 0.0)	Li_x_Ti_1−x_O_2_ NPs			
			x = 0.05,	x = 0.10	x = 0.15	x = 0.20
C_s_ (F g^−1^)	1.5 A g^−1^	513	666	822	747	579
	2 A g^−1^	468	592	764	664	512
	4 A g^−1^	408	520	688	552	448
	6 A g^−1^	360	456	612	492	408
	8 A g^−1^	304	384	544	432	352
	10 A g^−1^	260	320	480	360	290
	15 A g^−1^	210	240	390	300	249
(%) Capacity retention at 10 A g^−1^ after 5000 cycles		87.1	89.0	92.6	91.7	90.4
Energy density (W h kg^−1^) at 1.5 A g^−1^		64.12	83.25	102.75	93.38	72.37
Power density (W kg^−1^) at 1.5 A g^−1^		51.04	66.67	176.04	104.16	51.04
EIS analysis	R_s_ (Ω)	4.05	4.03	4.11	4.10	4.03
	R_ct_ (Ω)	0.86	0.80	0.56	0.43	0.31
	W_R_	2.95	2.86	2.34	2.72	2.74

**Table 3:** Electrochemical performance comparison of TiO_2_-based electrodes synthesized by different methods, performed in different electrolytes and current densities or scan rates.

Electrode	Synthesis method	Electrolyte	C_s_	Current density or scan rate	Capacity retention (number of cycle test)
AC/TiO_2_ electrode^7^	Hydrothermal method	0.5 M NaCl	515 mF/cm^2^	5 mV s^−1^	–
Homogeneous Co_3_O_4_/TiO_2_ nanotube electrode^16^	Chemical bath deposition (CBD)	2 M KOH	662 F g^−1^	1 A g^−1^	86% (4000)
TiO_2_ with Kapton tape, annealed under air^19^	Hydrothermal method	2 M KOH	57.62 F/cm^2^	10 mV s^−1^	90% (10,000)
Mn-TiO_2_ electrode^20^	Hydrothermal method	1 M Na_2_SO_4_	328 F g^−1^	5 A g^−1^	84% (2000)
N-doped TiO_2_ electrode^21^	Sol–gel method	3.0 M KCl	311 F g^−1^	1 A g^−1^	98.9% (4000)
Black TiO_2_ electrode^39^	Electrochemical self-doping	1 M Na_2_SO_4_	15.6 mF/cm^2^	100 mV s^−1^	96% (5000)
TiO_2_@C NRAs electrode^40^	Anodization process	0.5 M Na_2_SO_4_	23.6 mF/cm^2^	5 mV s^−1^	91% (1000)
The hollow N-TiO_2_ shells electrode^41^	Sol–gel method	1 M Na_2_SO_4_	2.48 mF/cm^2^	10 mV s^−1^	88.7% (1000)
Li_0.10_Ti_0.90_O_2_ NPs electrode ^This work^	Sol–gel method	0.5 M Li_2_SO_4_	822 F g^−1^	1.5 A g^−1^	92.6% (5000)

## Conclusions

In summary, anatase Li_x_Ti_1−x_O_2_ NPs (x = 0, 0.05 0.10, 0.15 and 0.20) could be synthesized by the sol–gel process. The anatase phase with space group I41/amd of tetragonal Li_x_Ti_1-x_O_2_ NPs was confirmed by XRD results. Additionally, the monoclinic phase of Li_4_Ti_5_O_12_ was detected in samples with x higher than 0.10, suggesting for the reduced conductivity and electrochemical performance of samples. Raman spectra demonstrated characteristic peaks that represented the anatase phase of TiO_2_ in all samples. Moreover, FT-IR spectra also confirmed the existence of different modes of vibration between Ti and O atoms in the TiO_2_ structure. TEM and FE-SEM images revealed the homogeneously dispersed Li-doped TiO_2_ NPs in a spongy like morphology with estimated particles size in a range 5–10 nm by TEM. Such a morphology was suggested to enhance the porosity, and to form an excellent conducting network of NPs for charges transfer. The BET results illustrated the increased surface area of Li_x_Ti_1−x_O_2_ NPs with increasing Li loading. Electrochemical studies showed the pseudocapacitive behavior of all samples with high-quality performance achieved in a sample of x = 0.10 that revealed the highest value of 822 F g^−1^ for specific capacitance at 1.5 A g^−1^, and could retain 92.6% of its original value after 5000 cycles test. Therefore, Li_0.10_Ti_0.90_O_2_ NPs with excellent performance in terms of high capacitance, high power density (176.04 W kg^−1^) and high energy density (102.75 W h kg^−1^) was suggested to be an appropriate material for supercapacitors electrodes application.
